# Analysis of root proteome unravels differential molecular responses during compatible and incompatible interaction between chickpea (*Cicer arietinum* L.) and *Fusarium oxysporum* f. sp. *ciceri* Race1 (Foc1)

**DOI:** 10.1186/1471-2164-15-949

**Published:** 2014-11-03

**Authors:** Moniya Chatterjee, Sumanti Gupta, Anirban Bhar, Dipankar Chakraborti, Debabrata Basu, Sampa Das

**Affiliations:** Division of Plant Biology, Bose Institute, Centenary Campus, P 1/12, CIT Scheme, VII-M, Kankurgachi, Kolkata, 700054 West Bengal India; Post Graduate Department of Biotechnology, St. Xavier’s College (Autonomous), 30 Park Street, Kolkata, 700016 India

**Keywords:** Chickpea (*Cicer arietinum* L.), *Fusarium oxysporum* f. sp. *ciceri* Race 1(Foc1), Defense response, Root proteomics

## Abstract

**Background:**

Vascular wilt caused by *Fusarium oxysporum* f. sp. *ciceri* Race 1 (Foc1) is a serious disease of chickpea (*Cicer arietinum* L.) accounting for approximately 10-15% annual crop loss. The fungus invades the plant via roots, colonizes the xylem vessels and prevents the upward translocation of water and nutrients, finally resulting in wilting of the entire plant. Although comparative transcriptomic profiling have highlighted some important signaling molecules, but proteomic studies involving chickpea-Foc1 are limited. The present study focuses on comparative root proteomics of susceptible (JG62) and resistant (WR315) chickpea genotypes infected with Foc1, to understand the mechanistic basis of susceptibility and/or resistance.

**Results:**

The differential and unique proteins of both genotypes were identified at 48 h, 72 h, and 96 h post Foc1 inoculation. 2D PAGE analyses followed by MALDI-TOF MS and MS/MS identified 100 differentially (>1.5 fold<, p < 0.05) or uniquely expressed proteins. These proteins were further categorized into 10 functional classes and grouped into GO (gene ontology) categories. Network analyses of identified proteins revealed intra and inter relationship of these proteins with their neighbors as well as their association with different defense signaling pathways. qRT-PCR analyses were performed to correlate the mRNA and protein levels of some proteins of representative classes.

**Conclusions:**

The differential and unique proteins identified indicate their involvement in early defense signaling of the host. Comparative analyses of expression profiles of obtained proteins suggest that albeit some common components participate in early defense signaling in both susceptible and resistant genotypes, but their roles and regulation differ in case of compatible and/or incompatible interactions. Thus, functional characterization of identified PR proteins (PR1, BGL2, TLP), Trypsin protease inhibitor, ABA responsive protein, cysteine protease, protein disulphide isomerase, ripening related protein and albumins are expected to serve as important molecular components for biotechnological application and development of sustainable resistance against Foc1.

**Electronic supplementary material:**

The online version of this article (doi:10.1186/1471-2164-15-949) contains supplementary material, which is available to authorized users.

## Background

Plants are often challenged by different types of biotic and abiotic stress factors. Their immobile nature precludes escape from these stress causing agents. Therefore, they possess preformed and inducible defensive strategies to overcome these stresses. In most cases, the host arrests the invading rival at the site of penetration [1]. Such immune response adapted by the host is termed as pattern triggered immunity (PTI) which include reprogramming of host cellular metabolism, reinforcement of cell wall by callose occlusions and production of antimicrobial compounds that act directly to prevent pathogen invasion [2, 3]. However, in some selected cases the invading pathogens secrete effector molecules that try to overcome host immunity, which in the absence of cognate host resistant protein/proteins (R-proteins) lead to effector triggered susceptibility (ETS) [4]. On the other hand, in the presence of cognate R protein/proteins the host mounts a defense response of much greater amplitude known as the effector triggered immunity (ETI), which largely overlaps with that of PTI [5]. However, the defense mechanisms of both PTI and/or ETI are regulated by altered protein synthesis and their time dependent degradation. Hence, qualitative and quantitative changes in protein levels are believed to be probable indicators of the ultimate outcome of any plant-pathogen interaction.

Amongst agronomical important crop plants, legume crops are known for their nutritive value that play very important roles in human nutrition as well as serve as supplement to improve growth of livestock [6]. Besides, they also fix atmospheric nitrogen enhancing soil fertility and boosting the yield of subsequently grown crops [7]. These crops are equally vulnerable towards pathogen. But studies on the molecular interaction involving legume-pathogen case study are significantly limited. Chickpea is the third most important legume crop in the world and the most important one in India (FAO). It is a rich source of digestible protein, and hence is considered globally as a valuable crop. However it is found that it accounts for 10 to 15% of yield loss worldwide by wilt causing fungus *Fusarium oxysporum* f. sp. *ciceri* (Foc). This seed or soil borne fungus has two different pathotypes, a yellowing pathotype and a wilt causing pathotype [8]. Amongst eight pathogenic races of Foc (Races 0, 1, 1B/C, 2, 3, 4, 5, and 6) Race 1, known to show a wide geographic distribution throughout India has received major scientific concern. The fungus invades the plant via roots, colonizes the xylem vessels and prevents the upward translocation of water and nutrients, finally resulting in wilting [9]. Yellowing of rootlets, chlorosis of basal leaflets and drooping of lower branches are the initial symptoms of pathogenic infection in chickpea plants [10]. Until recently, *Fusarium* wilt was being managed by resistance breeding programs. But the main hurdle faced by plant breeders was pathogenic variability and mutability that resulted in breakdown of natural resistance over prolonged period of time and generations [11]. Although, chemical fungicides are used as alternatives under such circumstances [12], but high cost and environmental safety issues are known to raise social concerns regarding its long term utilization. Therefore a proper understanding of the molecular mechanism involved in chickpea-Foc1 interaction could suggest effective measures for developing sustainable resistance.

Previous studies conducted on understanding the molecular interaction of chickpea- *Fusarium oxysporum* was based mainly on transcriptomic studies taking lead from the model plant *Arabidopsis thaliana* and tomato (*Solanum lycopersicum*) [13, 14]. Moreover, previously published histopathological reports suggested that Foc1 enters the roots through the breaches of root hairs and colonizes the xylem vessel of compatible host at about 4dpi (days post inoculation). Rapid establishment of Foc1 coupled with massive tissue disintegration led to the total collapse of root architecture ultimate causing wilting of susceptible plants at about 12dpi, whereas resistant plants showed minimal signs of stress even at later stages of infection [15–17]. Besides, reports based on transcriptomic studies suggested early recognition of wound inducing Foc1 by the host. Such early recognition triggered reprogramming of the primary metabolism of the host where ROS (reactive oxygen species), cellular transporters, transcription factors and sugar molecules acted as signal modulators [17, 18]. Apart from these, biochemical analyses and analytical studies on molecular markers and molecular linkages relating to wilt disease were also performed [19–21]. But the inferences drawn from transcriptomic studies are rather inadequate without proteomic support, as there are reports of huge numbers of genes with no assigned functions at their protein level. Additionally, the correlation between mRNAs and protein levels are remarkably low and fail to provide indications about post translational modifications or protein-protein interaction that are believed to have significant regulatory effect on defense responses [22]. Thus, in order to predict the actual scenario of pathogen driven molecular signaling within the host, the knowledge of defense responsive proteins are strongly desirable.

The present study involves understanding of chickpea-*Fusarium* interaction using proteomic techniques like two dimensional electrophoresis (2DE) and mass spectrometry (MALDI-TOF MS and MS/MS) followed by high throughput data base search. These techniques are effectively used nowadays to identify and analyze differentially expressed proteins involved in plant pathogen interaction and also their post translational modifications [23]. Barring a few, most of these proteomic studies are performed on model plants like *Arabidopsis* or *Medicago* [24, 25]. Information gathered from these model plants definitely boost up knowledge of plant immunity but biological interpretation of this knowledge in crop models require experimental substantiation. Moreover, some features and processes are likely to be unique for crop plants and hence cannot be approached via model plant in totality [26]. The present study focuses on the legume crop chickpea and its early response to infection by Foc1. This study aims to understand the mechanistic basis of susceptibility and/or resistance offered by two different genotypes (JG62 wilt susceptible, WR315 wilt resistant) respectively. Approximately 100 proteins were significantly identified by MALDI-TOF MS and MS/MS which included differentially regulated as well as unique proteins identified from both resistant and susceptible genotype of chickpea at different time points of 48 h, 72 h and 96 h after infection with Foc1. These identified proteins are categorized and their probable roles in plant defense are illustrated through interaction network based studies.

## Methods

### Plant growth and fungal treatment

Chickpea (*Cicer arietinum* L.) genotypes JG62 (wilt susceptible) and WR315 (wilt resistant), obtained from ICRISAT (International Crops Research Institute for Semi Arid Tropics), Hyderabad, India were used for experimental analysis. Seeds of both genotypes were grown in a mixture of soil and sand (1:1) under natural green house conditions of 22 to 28°C, 35 to 40% relative humidity and 16 h:8 h photoperiod of day and night respectively [15].

*F. oxysporum* f. sp*. ciceri* Race1 (Foc1) was obtained from ICRISAT and further purified according to the protocol of Summerell *et al* [27]. Spores obtained were harvested and stored at -80°C until further use. Two week old seedlings of both genotypes were inoculated with Foc1 using sick soil method as described by Gupta *et al* [15]. Plants of both genotypes grown on inoculum free soil served as control samples. Both control and infected plants were kept under same growth conditions. Root samples from control and infected plants at 48, 72 and 96 h post inoculation (hpi) were harvested, instantly frozen in liquid nitrogen and stored at -80°C for further analysis. Proteins were extracted from pooled tissue to run triplicate gels of each time points [28]. The entire experiment of plant growth and fungal treatment was repeated three times to generate three biological replicate.

### Protein extraction and quantification

Chickpea root proteins were obtained from one gram of root tissue by following Phenol-SDS buffer extraction method with sonication [29]. One gram of root tissue was pulverized in mortar and pestle with liquid nitrogen and homogenized with 3ml of SDS buffer (30% sucrose, 2% SDS, 0.1M Tris-Cl, 5% β-mercaptoethanol and 1 mM phenyl methyl sulfonyl fluoride (PMSF), pH 8.0). The extract was sonicated (60 amps, 15 secs, 6 times) and further treated with Tris buffered phenol. The phenolic phase obtained by centrifugation at 8000 g for 10 min at 4°C was rinsed with SDS buffer. This final phenolic phase was collected and precipitated overnight with four volumes of 0.1M ammonium acetate in methanol at -20°C. Precipitate was obtained at 10,000 g for 30 min.Washing of protein pellet was performed thrice at 8,000 g for 10 min with cold 0.1 M ammonium acetate and finally washed with cold 80% acetone. The pellet was then dried and resuspended in 100 μl sample buffer (Biorad) for further analysis. Extracted proteins were quantified using Bradford protein assay method using BSA as standard [30].

### Two dimensional polyacrylamide gel electrophoresis (2D-PAGE)

Isoelectric focusing (IEF) was carried out on PROTEAN IEF cell (Bio-Rad, USA) using immobilized pH gradient (IPG) strips. Two hundred fifty micrograms of each sample protein dissolved in 185 μl of rehydration sample buffer (8M urea, 2% CHAPS, 50 mM DTT, 0.2% Biolyte ampholytes) was loaded onto 11 cm immobilized pH 3-10 nonlinear (NL) gradient strips (Bio-Rad, USA) and was passively rehydrated overnight at room temperature. IEF was conducted at field strength of 600 V/cm and 50 mA/IPG strip. The strips were focused at 250 V for 20 min, 8000 V for 2 h 30 min with linear voltage amplification and finally to 20,000 Volt hour with rapid amplification. After focusing the strips were reduced and alkylated using 135 mM DTT and 135 mM iodoacetamide respectively, in 4 ml of equilibration buffer (20% v/v glycerol, 0.375M tris- Cl, 6M urea, 2% w/v SDS, pH8.8) for 15 min. Second dimensional electrophoresis was run with strips transferred to 12% SDS polyacrylamide gels (13.8 cm × 13.0 cm × 1 mm) in an AE-6200 slab electrophoresis chamber (Atto Biosciences and Technology, China) at a constant volt (200 V) for 3 h 30 mins in tris-glycine SDS running buffer. The gels were stained with 0.1% (w/v) coomassie brilliant blue R-250 (Sigma) overnight, destained and stored in 5% acetic acid at 4°C. 2D-PAGE gel separation was performed with both technical and biological replications of three.

### Image acquisition and analysis

Coomassie stained 2-D gel images were captured with Versa Doc Imaging system (Model 4000, Bio-Rad, USA) and analyzed with PD Quest Advanced 2-D gel analysis software (version 8.0.1, Bio-Rad, USA). For this study in total 72 reproducible gels were generated (three replicates, four time points, two genotypes and three biological replicates). Three technical replicates from three biological replicates at different time points (control, 48 h, 72 h, 96 h) for both genotypes (JG62,WR315) were assembled to create the master gel image (match set). Replicate gels used for making the match set had correlation coefficient value of at least 0.8. Background subtraction between the gels was done using floating ball method. Spots were detected automatically by the spot detection parameter wizard using Gaussian model with advance settings, by choosing faint spot, small spot and large spot cluster. Detected spots were visually checked and manually added when required [31]. Each spot included for analysis were present at least in two of the three replicate gels for a particular time point and also was of high quality. Detected spot volumes were normalized by the spot volume of the entire gel and used as a parameter for quantifying protein abundance. The differential spots which showed statistical significance level of p < 0.05 (Student’s t-test) were selected for analyses. However, the spots selected for downstream MALDI-TOF MS and MS/MS analyses fell under three main categories. Firstly it included the spots showing 1.5 fold changes (above or below) in protein abundance level in infected samples at least in any of the time points as compared to the comparable protein level of both the controls. Second category included spots which were accumulated after infection and present in more than one time point in infected samples but absent in controls. Third category included qualitative spots which are reproducibly present only in one infected variety for a particular time point. Spots which were present only in one replicate were not considered for analysis to minimize the interference of missing value. Experimental molecular mass and pI were calculated using 2D-PAGE gel images of standard molecular mass and pI markers. Data were further analyzed using Statistica v10.0 software (Statsoft Inc) through coefficient of variance calculation (CV), followed by comparison of control and treated values to find out statistical differences by multivariate analysis of variance (MANOVA) and Duncan’s multiple range test (DMRT), at *p* value 0.05. Protein spots that showed significant difference between treatments through DMRT were further processed for downstream MALDI-TOF MS and MS/MS analyses.

### Protein identification using MALDI-TOF MS and MS/MS

Protein spots were manually excised from 2D-PAGE gels, destained and in gel digested according to the protocol mentioned by Shevchenko *et al.* [32] with minor modifications. In gel digestion of proteins were carried out with porcine trypsin (Promega, USA) and peptides were extracted with 25% acetonitrile and 1% trifluroacetic acid. One microlitre of sample was loaded along with matrix (1 μl, α-cyano-4-hydroxy cinnamic acid, HCCA) (Bruker Daltonics, Germany) in an Anchor Chip MALDI Plate (Bruker Daltonics, Germany).

Mass spectra were generated in an Autoflex II MALDI TOF/TOF (Bruker Daltonics, Germany) mass spectrometer equipped with a pulsed nitrogen laser (λ-337 nm, 50 Hz) in the m/z range from 500 to 3500 Da. The enzyme used was trypsin with one missed cleavage. The spectra obtained were analyzed with Flex Analysis Software (version 2.4, Bruker Daltonics, Germany) for deletion of matrix peaks and tryptic autolysis peaks. Processed spectra were then searched using MS Biotools (version 3.2) program against the taxonomy Viridiplantae (Green plants) in the MSDB 20060831 (3239079 sequences; 1079594700 residues), NCBInr 20140323 (38032689 sequences; 13525028931 residues), SwissProt 2013_12 (541954 sequences; 192668437 residues) databases using MASCOT search engine (version 2.2). The standard parameters used in the search included peptide mass tolerance (±0.5 Da); fragment mass tolerance (±0.8 Da); proteolytic enzyme (trypsin); global modification (caramidomethyl, Cys); variable modification (oxidation, Met); peptide charge state (1+) and maximum missed cleavage of 1, for MALDI-TOF MS minimum S/N = 10 and for MS/MS minimum S/N =3. The significance threshold was set to a maximum of 95% (p</= 0.05). The criteria used to accept protein identification were based on molecular weight search (MOWSE) score, and the percentage of sequence coverage. From each samples most intense m/z values were chosen for further fragmentation (MS/MS). Automatic decoy database search was performed by choosing the decoy checkbox on MASCOT search engine. Decoy search was performed to avoid false identification of peptide by matching it to a random sequence from a decoy database. Only the results with 0% false discovery rate were accepted. Final protein identification was done by a combined search of PMF (Peptide Mass Fingerprint) and MS/MS data in MASCOT search engine.

### Protein interaction network generation and analysis

Pathway Studio software (version 7.1) (Ariadne Genomics, USA) and Res Net database (version 3.0) was used to study the biological interactions [33] between the identified proteins of the present study. These differential and unique protein sequences identified by PMF and MS/MS studies were subjected to BLAST analyses at TAIR database (The Arabidopsis Information Resource) and their homologous genes (bearing TAIR gene IDs, Additional file 1) used as inputs for network generation. Ambiguities and components without any interactive neighbors were eliminated from the import list. Interaction network was generated using the neighbor joining method with a degree of correlation as 1 (only the immediate upstream and downstream neighbors having direct relationship to the protein/protein products were considered for analyses). In addition, standard filter parameters and relation types were selected for interaction map generation. Presence of the identified proteins in known biological pathways was analysed using AraCyc and Ariadne Pathway data list. Functional classification of the identified proteins based on gene ontologies (GO) were also studied using Pathway Studio software. In both cases statistical significance (p < 0.05) of the pathway locations and GO classification of the identified protein were calculated.

### Quantitative real time pcr (qRT-PCR)

Total RNA was extracted from one gram root tissues of infected and uninfected plants of both genotypes at different time points of 48 h, 72 h and 96 h post infection. RNA was extracted using TRI reagent kit (Himedia, India) as per manufacturers’ instruction. For avoiding any DNA contamination RNA samples were treated with RNase free DNase (Fermentas, USA). cDNA synthesized using Revert Aid first strand cDNA synthesis kit (Fermentas, USA), was further used for qRT-PCR. Specific primers were designed based on the corresponding nucleotide sequence of identified proteins from CTDB (Chickpea Transcriptomic Database), DFCI (*Medicago trancatula* database), PDB (Protein data Bank) and NCBI database using Gene Runner software (version 3.1) and listed in Additional file 2. qRT-PCR was performed on Biorad i cycler (Bio-Rad I-Q5, USA) using SyBr green super mix. A reaction mix of 20 μl was prepared containing 25 ng cDNA, 0.3 μM of forward and reverse primers. The PCR conditions used were 95°C for 5 mins, followed by 40 cycles at 95°C for 30 sec, 50°C-55°C for 30 sec and 72°C for 30 sec [16]. A melt curve was also generated at the end of each PCR cycle to verify primer specificity. Sample variation was minimized by normalization using actin as internal standard [34]. Mean fold change was calculated using 2 ^-ΔΔct^ method [35]. All experiments were repeated three times and standard error was calculated.

## Results and discussion

### Analysis of chickpea root proteome

**Figure 1:**
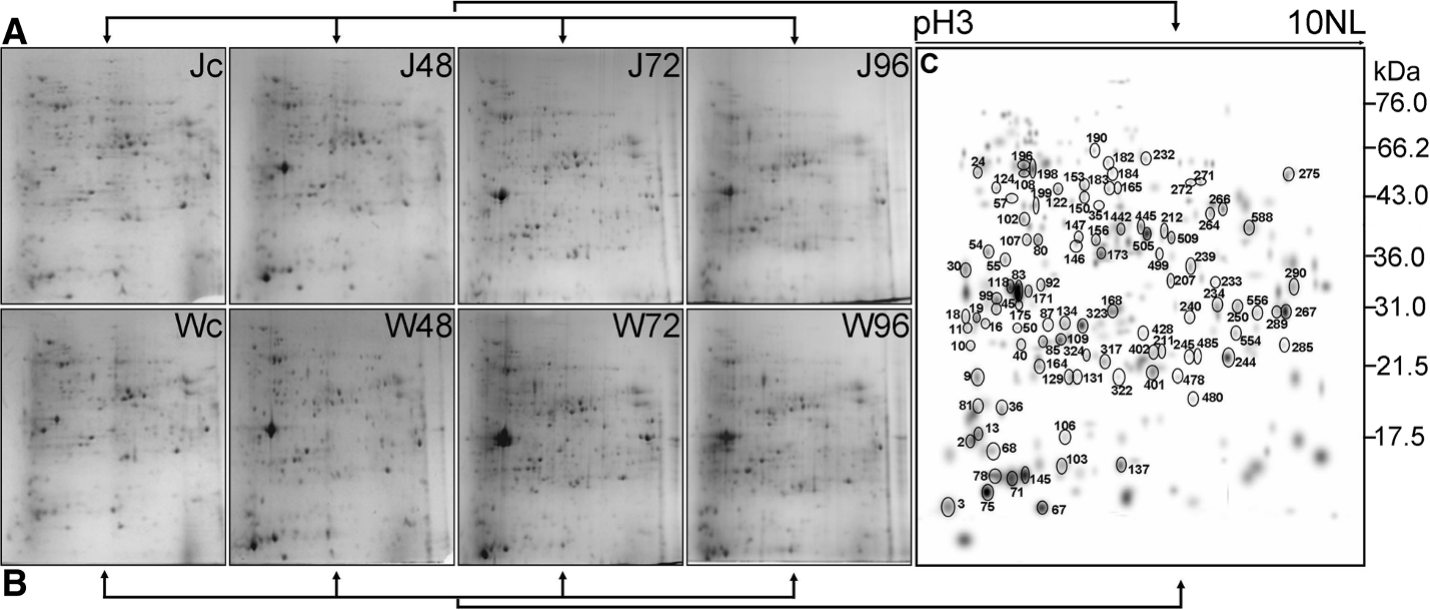
**Root proteome expression profile of control and Foc1 (** ***F. oxysporum*** **f.sp.** ***ciceri*** **Race1) infected chickpea genotypes JG62 (A), WR315 (B).** Root proteins (250 μg) of control (Jc) and infected JG62 extracted at different time intervals of 48 h, 72 h, 96 h (J48, J72, J96) post infection **(A)**. Root proteins (250 μg) of control (Wc) and infected WR315 extracted at different time intervals 48 h, 72 h, 96 h (W48, W72, W96) post infection **(B)**. Total proteins separated in first dimension (IEF,11 cm IPG strip, 3-10 NL) followed by second dimension in 12% SDS-PAGE, stained with coomassie brilliant blue R-250. Master gel generated by PD Quest **(C)**. Identified spots are encircled and the number corresponds to the spot number mentioned in Additional file 5.

Chickpea root proteome was studied with a view to understand the molecular mechanism governing the susceptibility and/or resistance of chickpea plant upon pathogen infection. Previous results based on histopathological and transcriptomic analyses performed by our research group as well as others, suggested the time points of 48 h, 72 h and 96 h to be crucial for delineating the early defense responses of chickpea during Foc1 attack [15, 16, 18]. These previous reports stated 96 h as the onset for xylem vessel colonization in compatible roots, while significant differential transcriptomic alterations were detected at as early as 48 h in both the susceptible and resistant genotypes [15, 16, 18]. An estimated protein yield for all the samples are provided in Additional file 3. Total root proteins were resolved onto 11 cm IPG strip (pH 3-10 NL). Figure 1(A and B) shows representative 2D experimental gel profiles corresponding to control and infected samples at different time points for both the genotypes, JG62 and WR315 respectively. The experimental design is shown in Additional file 4. Three independent experiments were performed to ensure that the changes in protein abundance at each time point were reproducible and significant. Two dimensional gel analyses indicated differential protein profiles for JG62 and WR315 plants upon Foc1 infection. Further PD Quest software analysis detected a total of 274 spots in the master gel (Figure 1C).The number of total spots detected and the differential spots (quantitative and qualitative) obtained post inoculation with Foc1 for each sample is provided in Additional file 3. To assess the reproducibility of the corresponding protein quantification, the CV was calculated for all protein spots, at all time points examined. The CV of protein spots for each sample type and time points was within 21% which is in accordance with other plant stress related studies [36] indicating stability and reproducibility of the present data. Among the total 206 differential spots obtained 163 spots which fell under the previously described three categories were processed for downstream MALDI-TOF MS and MS/MS analysis. MS/MS analyses was performed with 137 spots of which 100 spots that showed significant scores were taken into consideration for further functional clustering. Differential spots obtained due to differences in genotypes, depicting the natural variation between the susceptible and resistant genotypes (i.e differentially abundant between control samples of JG62 and WR315) were excluded from further downstream analyses in the present study (data not shown). Relevance of such differences between both genotypes that could also add significantly to the understanding of chickpea-Foc1 interaction shall be dealt separately in future studies. Selected protein spots were found to be interspersed at and around the median region of IPG strip suggesting the critical pH range for resolving the differential proteins to be around pH 4-7 (Figure 1C). Finally MS/MS analyses using mascot search engine in the available databases (NCBI, MSDB, Swissprot) led to the successful identification of 100 spots (Figure 1C). The details of these proteins and their peptides identified by MS/MS is provided as a table in Additional file 5. Among these 100 spots, 65 spots showed significant (1.5 fold change) quantitative changes in infected genotypes (JG62 and WR315) as compared to comparable protein level in control and 35 spots showed qualitative changes. Out of these 35 spots, 28 were accumulated after infection in more than one time point of either/or both infected genotypes, absent in controls and 7 spots were unique for any one time point and genotypes. MANOVA followed by DMRT indicated the statistical significance of the data provided in Additional file 6. Means that do not share any common alphabet differ significantly by DMRT at 5% level.

### Identification and classification of differential and unique proteins in chickpea during Foc1 infection

**Figure 2:**
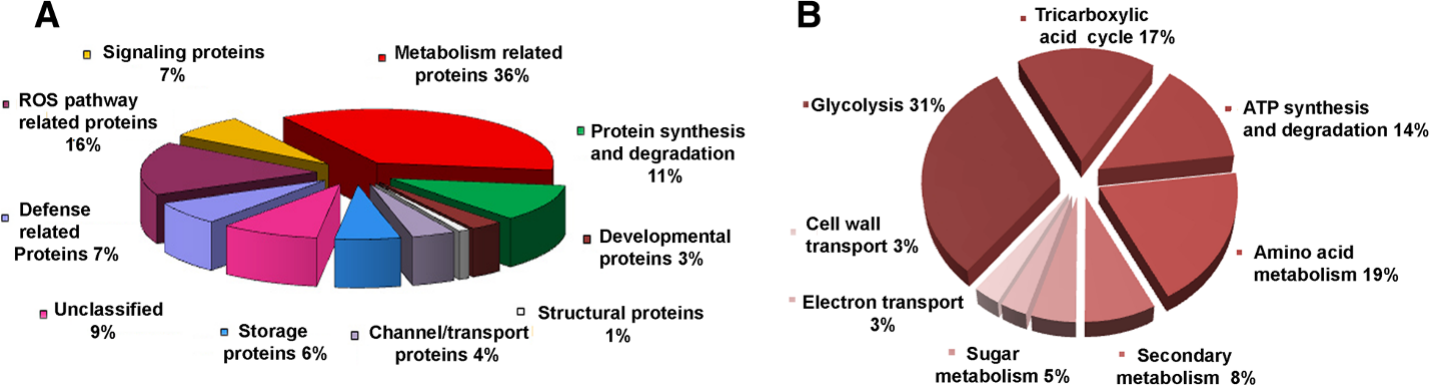
**Distribution of functional classification of Identified proteins.** Functional classification and relative distribution of proteins altogether identified in JG62 and WR315 chickpea genotypes after infection **(A)**. Classification and categorization of metabolism related proteins **(B)**. The proteins belonging to different categories and their expression level at different time intervals are mentioned in Additional file 5 in detail

The identified proteins were classified into nine functional categories based on their putative biological functions and proteins with unassigned functions were categorized as unclassified group. Metabolism related protein (36%) constitute the most abundant group followed by proteins related to scavenging of reactive oxygen species (ROS) (16%), protein synthesis and degradation related proteins (11%), defense related proteins (7%), signaling proteins (7%), storage proteins (6%), transport proteins (4%), developmental proteins (3%), structural proteins (1%) (Figure 2A). The unclassified group accounts for 9% of total identified proteins. Metabolism related proteins were further classified into glycolysis related proteins (31%), proteins of TCA cycle (17%), ATP synthesis and degradation regulating proteins (14%), proteins related to amino acid metabolism (19%), secondary metabolism (8%) and sugar metabolism (5%). Moreover, 3% proteins were found to be related to electron transport and another 3% were related to cell wall metabolism and transport (Figure 2B). Many defense related proteins identified had scores below 70. Complete draft genome sequence of chickpea has been recently reported, but functional annotations of genes and gene products are still at initial stages. Chickpea is a legume and its closest completely sequenced neighbor legumes are model plants *Medicago* and *Lotus*. However, the functional annotation of these neighbor model legumes are also underway and constantly being updated. Besides, chickpea being a crop legume is expected to have some distinct differences with these model legumes. Such differences are likely to be reflected in the protein identification scores of chickpea when subjected to homology matches with these model legumes. Hence, all the important defense related proteins obtained from the present study were discussed even though their scores were in the range of 40-60. Previous studies conducted with chickpea also reported similar identification scores for protein identification [36, 37]. In most of the cases each protein spots were identified as a single, unique protein but in some cases the identified protein spots contained more than a single protein; in such cases, the first hit with maximum score was considered for their protein IDs [38]. In addition to this, multiple spots were also found which were identified as the same protein. The appearance of such proteins probably suggests them being chemically and/or molecularly different products of a single gene and referred to as protein species [39] (Additional file 5). They basically fall under three main categories (i) with same molecular mass and different pI; for example, Kunitz proteinase inhibitor (sp 2, sp 13), Annexin (sp107, sp 499), Glyceraldehyde 3 phosphate dehydrogenase EC 1.2.1.9 (GAPC) (sp505, 509), (ii) with different molecular mass but same pI; for example Cysteine proteinase (sp 99, sp 45),( iii) or with different molecular mass and pI; for example, Superoxide dismutase EC 1.15.1.1 (sp 67, sp103, sp401), Triose phosphate isomerase EC 5.3.1.1 (sp 109, sp 40, sp 85). The differences in Mr and pI values, suggest that these changes in the proteome are probably due to the post-transcriptional modification. They may belong to different members of the same functional family, indicated by small shift in the pI or are degraded protein products as suggested by significant differences between theoretical and observed Mr values. The slight differences in pI and Mr values probably reflect post translational modifications (like phosphorylation, acetylation, glcosylation, methylation) occurring *in vivo* or may be the result of modifications such as deamidation of the proteins during sample preparation and processing [25]. It is known that the same protein may have different functions in different subcellular compartments. In the present study superoxide dismutase (sp 103,sp 401), triose phosphate isomerase (sp 109,sp 85) and GAPC (sp 505, sp 509) were identified as protein variants present in different cellular compartments like mitochondria, chloroplast or cytosol. Hence their multiple forms may be attributed to their multiple cellular locations [39]. In most of the stress related studies GAPC showed post translational modification like phosphorylation and was found to be present as multiple protein species. But whether the same observation in the present study indicates same modifications needs validation [40].

### Proteins related to direct defense responses against Foc1

**Figure 3:**
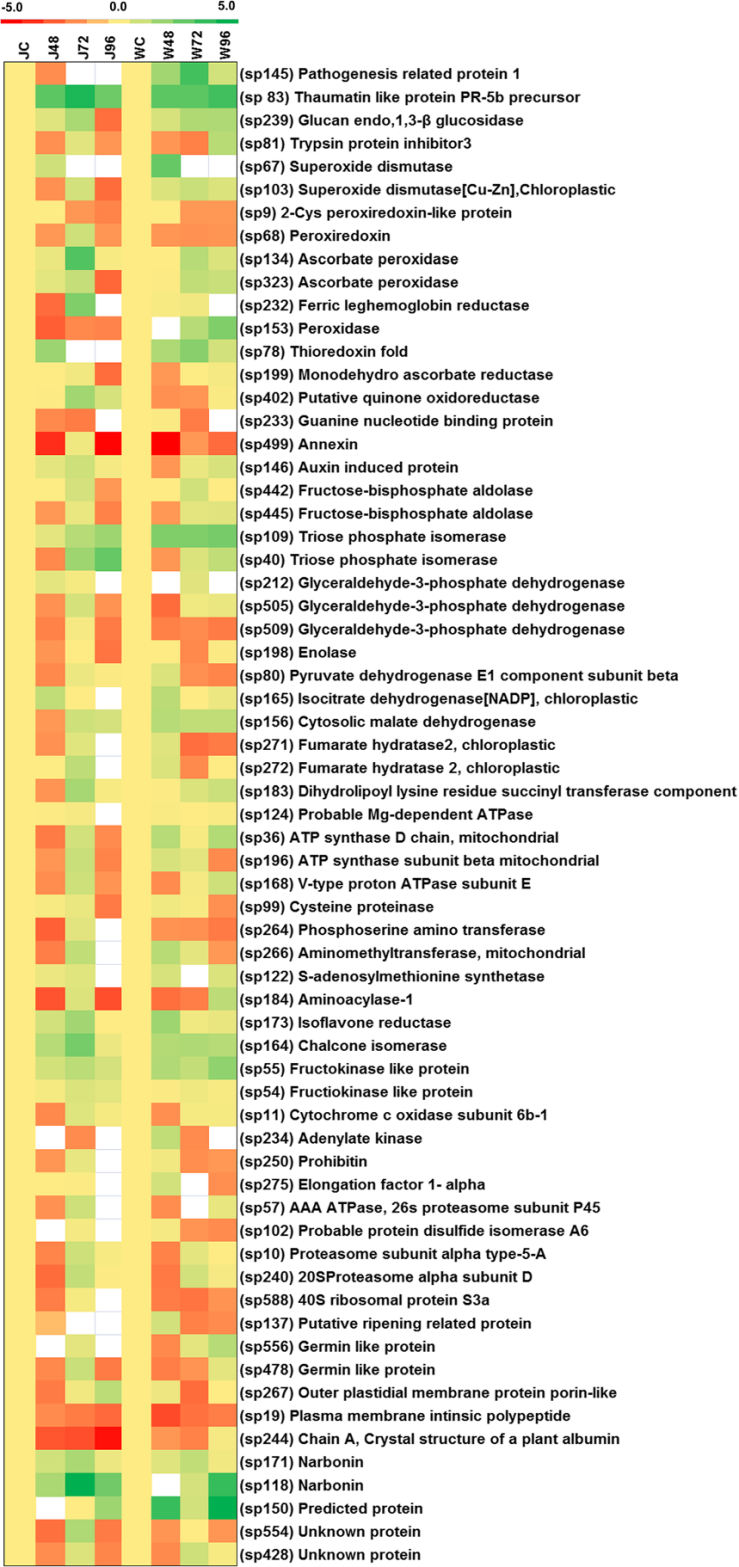
**Heat map representation of differentially expressed proteins of JG62 and WR315 chickpea genotypes on infection with Foc1.** Heat map was generated with the fold change values considering infected/control ratios. Each column represents a particular time point of infection and each row represents corresponding proteins with their identities. Up regulation or down regulation is indicated by the above scale which shows pale to saturated colors of green and red respectively. Yellow color represents mid-value and white represents no expression.

**Figure 4:**
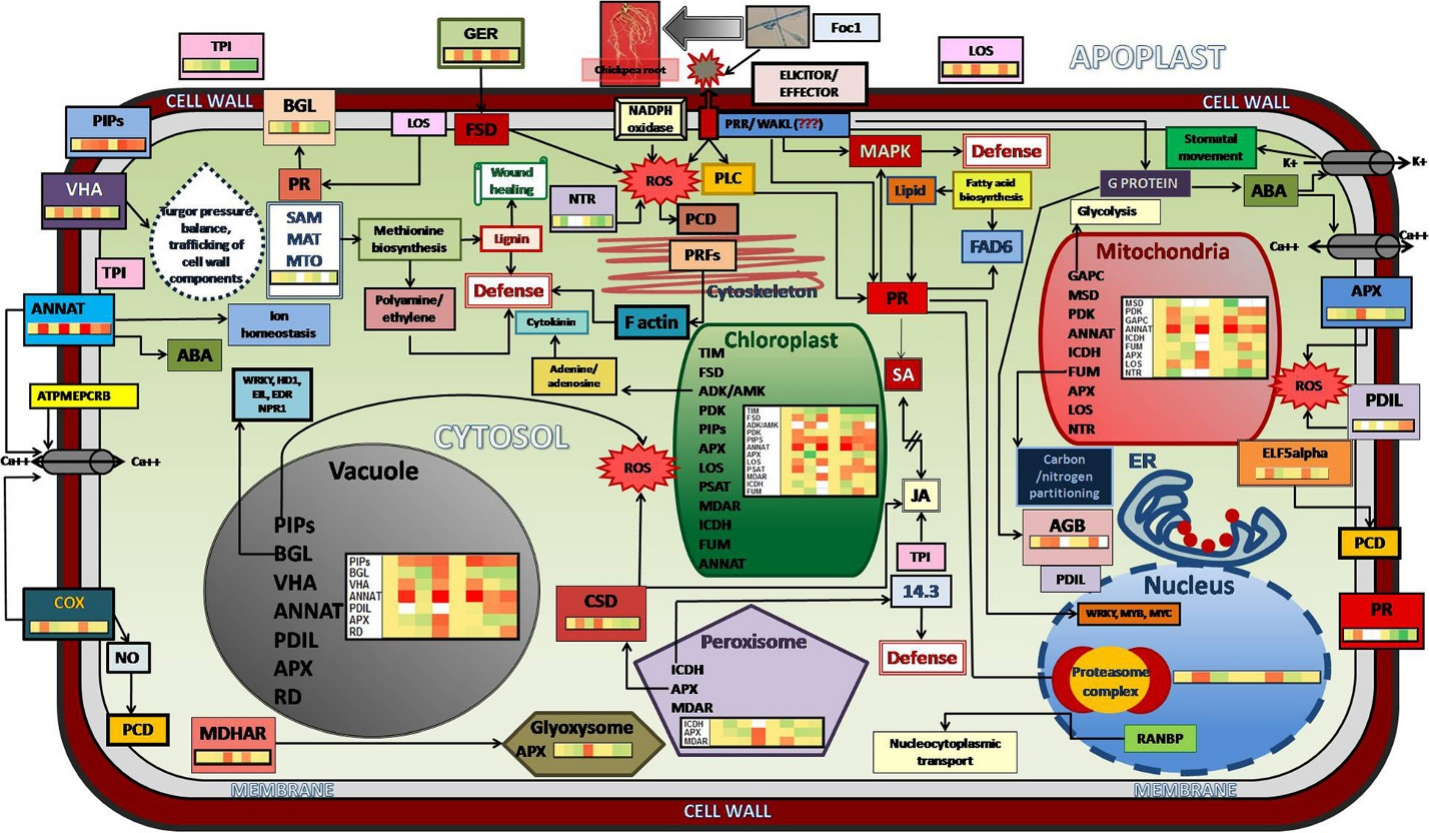
**Schematic representation showing the location and interaction between the different Foc1 induced proteins in chickpea roots.** Representation shows the intra and inter relationship between the Foc1 induced proteins and their regulatory biological processes. (Complete names of abbreviated proteins are provided in Additional file 1).

Defense related proteins contribute to about 7% of total identified proteins. They include PR1 (pathogenesis related protein 1), BGL, EC 3.2.1.39 (glucan endo 1-3 beta glucosidase), TLP (thaumatin like protein) and TPI (trypsin protease inhibitor) (Figure 3, Additional files 5 and 7). Pathway analysis showed the association of these proteins with defense and hypersensitive response related pathways (Additional file 8). Gene ontology (GO) based classification showed their relation with biological processes, molecular function and their cellular location (Additional files 9 and 10). Schematic network showed the interaction of these components with other Foc1 inducible proteins (Figure 4). PR1(sp 145) protein known to be directly involved in plant defense against pathogen attack was found to be accumulated at 48 h and 72 h post infection in resistant plants while in case of susceptible plants protein level was not detectable after infection (Figure 3, Additional files 5 and 7). PR1 expression known to be regulated by salicylic acid (SA) is positively regulated by NPR1 (Non expressor of PR genes1) during defense [41]. Besides, ACD (accelerated cell death), known to accelerate cell death in *Arabidopsis* is also a positive regulator of PR1 [42]. MAP kinase (Mitogen activated protein kinase), EDS4 (Enhanced disease susceptibility 4), PAD2 (Phytoalexin deficient 2) linked to fungal defense response also regulate PR1 expression [43, 44]. On the other hand, studies conducted on *Arabidopsis thaliana* reported EDR2 (Enhanced disease resistance 2), NPR3 and NPR4 to be negative regulators of PR1 [45, 46]. PR1 expression is also reported to be altered by phospholipase C and fatty acids [47, 48]. In the present study the increase of PR1 protein in resistant plants suggests its direct role in Foc1 induced defense, although the role of SA in modulating resistance in the present case study is still speculative. BGL also known as PR2, are enzymes which mainly act by hydrolyzing 1-3 β D glucosidic linkage of fungal cell wall and hence known to provide resistance in plants. BGL (sp 239) was found to be up accumulated in response to fungal attack in both genotypes. However, the susceptible plants showed highest accumulation at 72h (Figure 3, Additional files 5 and 7) that decreased later. Both BGL and PR1 are known to have SA dependent expressional regulation [49]. Both PR1 and BGL are reported to be upregulated in over expression lines containing EIL (ethylene-insensitive3-like) transcription factor in *Vigna mungo* indicating a positive role of ethylene in regulating defense response [50]. TLPs are pathogenesis related proteins having antifungal activity. TLP, also known as PR5 (sp 83,129) was found to be significantly increased in response to Foc1 in both genotypes (Figure 3, Additional files 5 and 7). However, in resistant plants it showed uniform accumulation while in susceptible plants (sp 129) it was found to be absent at later time points (72 h and 96 h). TLP was found to be up accumulated in *Medicago trancatula* during *Orobanche crenata* infection indicating that it may eventually take part in defense mechanism against parasitic infection [25]. TPI are known to participate in the wound induced defense response of plants against herbivores and pathogens. TPI (sp 2, 13, 81) were found to be uniformly enhanced in response to Foc1 induction in resistant plants while susceptible ones showed protein level undulations (except for sp 81, which showed uniform protein accumulation). (Figure 3, Additional files 5 and 7). TPI is positively regulated by JA signaling [51]. WRKY transcription factors coordinating herbivory are also known to regulate TPI expression [52]. The induction of TPI probably indicates the involvement of SA/JA mediated hormonal crosstalk which needs further experimentation. Role of PR proteins (PR1, PR2 and PR5) in modulating defense network were also elaborated by transcriptomic as well as proteomic studies involving wheat (*Triticum aestivum* L.) and stripe rust fungus *Puccinia striiformis* f.sp. *tritici* Eriks. (*Pst*) [53, 54].

### Role of ROS scavengers/regulators

Sixteen percent of total proteins were classified as ROS scavengers/regulators. Superoxide dismutases, EC 1.15.1.1 (SOD), Peroxiredoxin proteins, Ascorbate peroxidase, EC 1.11.1.11 (APX), Ferric reductase EC 1.6.2.6, Glutathione S transferase, EC 2.5.1.13 (GST), Peroxidase, Thioredoxin (NTRA, NTRB), Monodehydroascorbate reductase, EC 1.6.5.4 (MDHAR, MDAR), Quinone oxidoreductase, EC 1.12.5.1 etc (Figure 3, Additional files 5 and 7) are the proteins included in this class. Pathway analysis showed association of some proteins (SOD, APX, NTRA and NTRB, MDHAR and MDAR etc) with ROS regulatory pathways (Figure 4, Additional file 8). GO classification illustrated the roles of these proteins according to their biological processes, molecular functions and cellular components (Additional files 9 and 10). Figure 4 showed their cellular location and their interaction with other Foc1 induced proteins. SODs (sp 67, 103, 401) showed oscillations in protein accumulation post infection in both genotypes (Figure 3, Additional files 5 and 7). SODs are known to provide the first line of defense to infected hosts by scavenging the pathogen triggered ROS [55]. SODs are also reported to induce ROS mediated PR1 expression in *Nicotiana* [56]. APX (sp 134, 87, 323) (Figure 3, Additional files 5 and 7) is an important enzyme participating in anti oxidation metabolism in plants [57]. Besides, they are also reported to be upregulated during heat stress in *Arabidopsis* [58]. Differential induction of SOD and APX in the present study indicated the role of Foc1 induced ROS in triggering defense responses in chickpea. These observations support previous reports based on transcriptomic studies [16, 18]. Ferric reductase plays an important role in maintaining iron homeostasis, disruption of which may lead to generation of toxic free radicals. Ferric reductase (sp 232) also known to be an antioxidant for peroxides, showed enhanced protein level in susceptible plants compared to resistant ones (Figure 3, Additional file 5). GST (sp 324,211,317) showed marginal changes in protein accumulation in resistant plants while susceptible plants showed relatively sharp increments and decrements in protein level post Foc1 induction (Additional files 5 and 7). GSTs are reported to reduce oxidative stress inductive organic hydroperoxides in *Nicotiana benthamiana* following *Colletotrichum destructivum* infection [59]. In the present study, steady state protein level of GST in resistant plants may indicate lesser accumulation of oxidative stress components as compared to susceptible plants. NTR (sp 78) showed increment in resistant plants following Foc1 infection while susceptible plants showed sharp decline after 72 h of infection (Figure 3, Additional file 5). Such up accumulation of NTR only in resistant plants indicated their efficient role in regulating oxidative stress tolerance [60]. Previous proteomic studies conducted on wheat showed enhanced accumulation of GST and NTR during incompatible interaction with *Puccinia striiformis* f.sp. *tritici* Eriks. (*Pst*). Besides, level of peroxiredoxin was also found to be induced [54]. In addition transcriptomic studies showed the enhancement of peroxidase transcripts in wheat following *Puccinia striiformis* f.sp. *tritici* Eriks. (*Pst*) infection [53]. MDHAR (sp 199) showed similar protein accumulation levels in both plants post infection (Figure 3, Additional files 5 and 7). Such increment indicated role of MDHAR in JA mediated antioxidation metabolism in the present case study that was found to be similar to previous results reported on *Arabidopsis thaliana* [61]. Besides, increment of MDHAR also linked to increased lipid peroxidation which is marked as a feature during pathogen mediated membrane injury [62]. Quinone oxidoreductase, known to act as detoxifier of ROS induced oxidative stress along with GST was found to be up accumulated at later time points of infection in susceptible plants compared to resistant ones (Figure 3, Additional file 5).

### Role of signaling proteins

Signaling proteins constitute about 7% of total identified proteins. Guanine nucleotide binding protein (AGB), Annexins (ANNATs), ABA responsive protein (RAB), Ran binding protein (RANBP), Auxin induced protein and Zinc binding dehydrogenase are classified under this category (Additional file 5). Pathway analysis based on *Arabidopsis* homologues showed only the association of AGB with signaling pathway (Figure 4). While GO classified all the proteins in this category (AGB, ANNATs, RAB18 and RANBP) according to their relation with biological processes, molecular function and cellular components (Additional files 9 and 10). Network map showed their interaction with other Foc1 induced proteins (Additional file 8). AGB (sp 233) coupled with other G proteins and GPCRs are known to modulate defense responses in *Arabidopsis* [63]. Besides, AGB are also known to modulate ABA driven K^+^ and anion channels thus regulating stomatal movement [64]. In the present study, similar protein accumulation pattern of AGB (Figure 3, Additional file 5) in both plants indicate a common regulation of AGB that is probably directed towards stomatal movement, a significant phenomenon observed during vascular wilt. Annexins (sp 107, 499) (Figure 3, Additional files 5 and 7) are reported to regulate pH mediated cellular responses that are directly influenced by ABA and calcium conductance during stress in *Arabidopsis* and *Zea mays* [65, 66]. The up accumulation of annexins in both plants probably directs the role of Foc1 in triggering pH alterations as well as ABA driven calcium oscillations during infection that needs to be investigated. RAB (sp 71) was found to be up accumulated only in resistant plants post infection. RAB was reported to be induced during ABA perception that activated calcium influx in *Arabidopsis thaliana* suspension culture cells [67]. Such induction was further known to be mediated by phospholipase D activation [68]. In the present study induction of RAB only in resistant plants directs towards role of ABA and calcium signaling in modulating defense in chickpea during Foc1 infection. RANBP (Sp16) known to regulate nucleocytoplasmic transport under the control of hormones and light, was found to be uniquely expressed at 72 h post infection in susceptible plants [69]. The relevance of such selective induction in the present study requires further investigation.

### Role of metabolism related proteins

Majority of the proteins identified (36%) fell under metabolism related proteins (Figure 2A). This category was further re-categorized into several sub classes (Figure 2B, Figure 3, Additional files 5 and 7). Such large assemblage of metabolism related proteins indicates that pathogens usually target the host metabolism for self survival and reproduction, while on the other hand host puts forth complete effort in shielding their primary metabolism from the devastations of pathogen attack [16]. Pathway analysis showed the association of some of these proteins with metabolic pathways (Figure 4). GO classification grouped them according to their biological processes, molecular functions and cellular components (Additional files 9 and 10). Interaction map further showed the location and interaction of some of these proteins with their neighbors as well as within themselves (Additional files 8). Glycolytic enzymes triose phosphate isomerase, EC 5.3.1.1 (TIM) (sp 109, 85, 40) and glyceraldehyde dehydrogenase phosphate, EC 1.2.1.9 (GAPC) (sp 212,505,509) were found to show similar pattern of protein level undulations in both compatible and incompatible interaction suggesting the common role of glycolytic ATP on pathogen triggered immune response of host [70]. However, enolase EC 4.2.1.11 (LOS) (sp 198) showed sharp decline at later time points of infection in susceptible plants while resistant plants showed steady state protein level (sp 198) or sharp induction (sp 182, 351) at different time points of infection. Enzymes of TCA cycle such as isocitrate dehydrogenase, EC 1.1.1.42 (ICDH) (sp 165), malate dehydrogenase, EC 1.1.1.37 (sp 156) and fumarase, EC 4.2.1.2 (FUM) (sp 271,272) showed elevated or stable protein accumulation in resistant plants as compared to susceptible plants suggesting a constant energy supply, which is required for different processes like photosynthesis, respiration and photorespiration during stress [71–73]. ATP synthase (sp 36, sp 196) and ATPases (VHA) (sp 480,124,168) however showed similar protein level patterns in both plants after infection. This may indicate the need for maintaining energy and solute homeostasis necessary for protein sorting and cell wall repair that probably aid to cell protection during pathogen progression [74]. Similar interaction studies involving wheat and stripe rust fungus reported the increment of ATP synthase both at transcriptomic as well as proteomic levels [53, 54]. Cytochrome c oxidase, EC 1.9.3.1 (COX) (sp 11) which is known for translocation of protons to drive aerobic respiration as well as to regulate stress mediated signals [75] showed elevated protein levels in resistant plant at later time period as compared to susceptible plants. These findings suggest that even though, energy requirement is necessary for both the genotypes during stress, but proper channelization of energy needed for running basic metabolic activities controls resistance, which perhaps is efficiently maintained by the resistant plants. Cysteine protease (RD) (sp 45) showed up accumulation of protein only after Foc1 infection in both plants. RD is known to be important players in plant immunity, especially in regulating resistance response against necrotrophic pathogen [76]. In the present study the selective up accumulation of RD after Foc1 infection predicts the role of RD in regulating biotrophic interaction also. However, such assumption requires further experimental support. Phosphoserine amino transferase, EC 2.6.1.52 (PSAT) (sp 264) was found to be absent at 96 h in susceptible plants while resistant plants maintained a moderate protein accumulation level even after infection suggesting the need of serine biosynthesis which is known to be associated with photorespiration [77]. S-adenosyl methionine synthetase, EC 2.5.1.6 (SAM) (sp 122) a direct product of methionine catabolism acts as substrate for several transmethylation reactions including those that occur during lignin biosynthesis [78]. SAM was found to be absent at 96 h post inoculation in susceptible plants while resistant plants regained the protein accumulation at 96 h suggesting the role of transmethylation and lignin biosynthesis in somehow regulating repair mechanisms caused by pathogen invasion. Proteins related to secondary metabolism (sp 207, 173, 164) showed differential abundance level post infection in both plants. They are known to regulate defense response during biotic stress [79]. Methylesterase (sp 290) were found to be selectively enhanced at 72 h post infection in both the plants. Methylesterases are known to be directly or indirectly associated with defense reactions by regulating the degree of methyl esterification of pectin that is known as essential cell wall components [80]. Selective accumulation of methyl esterase after infection in both plants suggests a possible cell wall repair mechanism to be operational, which however may be more efficient in resistant plants as indicated by its elevated level.

### Role of proteins involved in its folding, synthesis and degradation

This group of proteins accounts for about 11% of total identified proteins. Pathway analysis showed the association of adenylate kinase EC 2.7.4.3 (ADK, AMK2) with protein synthesis and purine biosynthetic pathways (Figure 4). While all other proteins related to protein synthesis, folding and degradations showed enlistment under categories of GO (biological function, molecular function and cellular component) (Additional files 9 and 10). Network analyses also showed the intra and inter relationship of these proteins with other Foc1 induced proteins (Additional file 8). 26S proteasome subunits are known to contribute to both basal defense as well as *R* gene mediated defense in *Arabidopsis*. Activation of these proteins are known to regulate innate immunity both positively and negatively as appropriate protein degradation are necessary for mounting defense [81]. Besides, studies on *Nicotiana* reported the induction of 20S proteasome subunits that was found to be linked to HR and SAR [82]. In the present study the differential accumulation of 26S proteasome subunits EC 3.4.25.1. (sp 57, 10, 240) in both the plants post infection suggests the role of protein degradation in regulating defense (Figure 3, Additional files 5 and 7). However, whether such regulation is directed towards positive and/or negative influences needs to be investigated in detail separately for both compatible and incompatible interaction. Adenylate kinase EC 2.7.4.3 (ADK, AMK2) (sp 234) was found to be up accumulated in 48 h in resistant plants while susceptible plants maintained an overall low protein level. ADK, known to be involved in salvage pathways of adenine and adenosine also convert cytokinin and ribosides to corresponding nucleotides. Such cytokinin conversion regulates the hormonal level of plants [83]. Absence of ADKs is known to cause chloroplastic deformity in *Arabidopsis* [84]. In the present case study overall down accumulation of ADKs (Figure 3, Additional files 5 and 7) probably indicates pathogen mediated chloroplastic damage and hormonal alteration. Eukaryotic translation initiation factor (elF5lpha) (sp 106) protein was uniquely accumulated at 48 h in resistant plants while susceptible plants showed no accumulation. Studies conducted on *Arabidopsis* showed the involvement of elF5alpha in controlling resistance by preventing pathogen growth and development of *Pseudomonas syringae* [85]. Besides, elF5alpha was also up accumulated during infection with stripe rust fungus in resistant wheat plants [54]. However, whether the accumulation of elF5alpha protein at a specific time point post infection in resistant plants has a similar role in restricting the pathogen progression needs to be experimented. Protein disulphide isomerase (PDIL) (sp 102) was found to be up accumulated at 48 h in resistant plants which gradually declined at later time points however maintaining a moderate level compared to control plants even after 96 h of infection. In susceptible plants the accumulation level of PDIL were greater compared to control samples only at 72 h post infection and absent in other time points (Figure 3, Additional files 5 and 7). PDIL acts as chaperones of cysteine proteases, thus regulating their trafficking from endoplasmic reticulum to vacuole prior to PCD [86]. Besides, PDIL are also known to be reduced by thioredoxin reductases and actin and removing aberrant disulphides formed by oxidative stress [87]. Level of PDIL was found to be elevated in wheat following inoculation with stripe rust fungus [54]. The abundance of PDIL in resistant plants in the present case study suggests the operation of antioxidant defense machinery during incompatible interaction in chickpea against Foc1 attack.

### Role of developmental, structural, channel and storage proteins in Foc1 induced defense

Developmental, structural, channel and storage proteins contributes to about 14% in total.

Functional classification identified developmental proteins such as ripening related protein (RLP) (sp 137) and germin (sp 556, 478) to be differentially expressed in both the plants after infection (Figure 3, Additional files 5 and 7). RLP contains the conserved Bet v fold domain also present in major latex proteins (MLPs) and PR10 group of allergen proteins. These proteins are associated with fruit and flower development as well as defense. However their role in defense is not well characterized [88]. In the present study the protein abundance of RLP at 48 h in resistant plants suggests this protein somehow modulate initial defense response which requires further characterization. Germins, known to have roles in plant development and defense, are associated with extra cellular manganese-SOD activity [89]. The up accumulation of sp 556 protein at later time points (96 h) in resistant plants and protein level undulations of sp 478 in both plants post infection suggests a differential operation of antioxidant defense mechanism in controlling pathogen invasion in both plants. However, transcriptomic based studies reported the increment of germin like transcripts in response to stripe rust fungus specifically in resistant genotypes of wheat [53]. Structural protein profilins (PRFs) are actin monomer binding proteins that regulate the assembly-disassembly of uncapped-capped actin molecules in forming cytoskeletal filaments [90]. Profilin (sp 3) protein was found to be uniquely accumulated at 48h in resistant plants post infection. Such selective accumulation probably indicated the need of cytoskeletal assembly to strengthen the cell and prevent further fungal ingress. However, such assumption needs further experimental support. Channel proteins porin (sp 267) and plasma membrane intrinsic protein (PMIP/PIP) (sp 19) belong to the aquaporin family of proteins that are known to regulate hydraulic conductance during cold and oxidative stress [91]. The present study showed differential protein accumulation profiles in both genotypes after Foc1 induction, which suggested that probably the channel proteins regulated water transport differently during incompatible and compatible interaction. The enhanced level of these proteins at 96 h post infection in resistant plants compared to susceptible ones suggested proper water conductance in resistant plants when susceptible plants succumbed to wilting symptoms. Plant albumins are known to serve as storage proteins as well as defense responsive proteins possessing insecticidal and antimicrobial properties that are induced in response to stress [92]. The present study showed up accumulation of albumin (sp 244, 485) in resistant plants compared to susceptible ones indicating the role of storage proteins in controlling defense against fungal attack.

### Unclassified proteins

This group mainly includes proteins with unknown functions (Figure 3, Additional file 5). They contribute to about 9% of total proteins identified (Figure 2). Recent availability of chickpea whole genome sequences and updating of functional annotations is believed to provide proper naming and functional designations to these unclassified proteins [93].

### Probable roles of identified proteins in imparting resistance against Foc1

To understand the mechanism of resistance in plants it is important to know what are the different proteins involved and how they come into play during the pathogen attack. Pathogenesis related proteins are defense related proteins which are induced on pathogen attack and have a direct role in plant defense, but how these proteins operate or accumulate in compatible and incompatible interaction actually decides the sustainability of resistance. In the present study three important PR proteins were identified, PR1 (pathogenesis related protein 1), PR5b (Thaumatin like protein), PR2 (β-1, 3-glucanases) and their accumulation at different time points post infection were studied. All these PR proteins were found to show a stable level of accumulation in resistant plants after infection where as in susceptible interaction although the proteins appear in early time points but at later time point they either decrease or disappear (Additional file 5). More specifically PR1 which has antifungal activity showed high level accumulation in resistant chickpea plants post infection (Figure 3, Additional file 7). Both PR1 and PR2 are also known to be associated with salicylic acid and ethylene signaling indicating their probable roles in modulating defense [49, 50]. In addition, uniform accumulation of TPI (trypsin protease inhibitor) in resistant plants pointed towards the role of JA in regulating defense [51]. PR5b (Thaumatin like proteins) are known to be induced exclusively in response to wounding or pathogen infection. This protein exhibit a balanced accumulation in resistant plant which indicated its role in disease resistance (Additional file 5). This protein is known to inhibit hyphal growth and reduce spore germination probably by altering membrane permealization or by interacting with pathogen receptors [94]. PR2, involved in cleavage of the β-1, 3-glucosidic bonds of β-1, 3-glucan of fungal call wall, was found at elevated level post infection in susceptible plants which decreased at later time point (Additional file 5). This probably indicated an initial struggle between susceptible plants and pathogen, which was followed by pathogen overpowering the host. However in resistant plants a high and stable PR2 protein accumulation (Additional file 5) indicated that oligosaccharides from fungal cell wall probably acted as an activator, for other PR proteins or antifungal compounds, such as phytoalexins [95]. Besides, this study also mentions high level accumulation of a developmental protein namely ripening related protein (RLP) in incompatible interaction after infection (Additional file 5). Ripening related proteins are found to share homology with some defense responsive proteins in plants. Defense responsive proteins are often found to express during fruit ripening, suggesting that both these processes possibly share a common regulator [96]. Ethylene acts as a key regulator in fruit ripening and in response to stresses caused by pathogens and wounding [97]. Moreover accumulation of S-adenosyl methionine synthetase at later time period in resistant plant also indicates a role of ethylene in plant defense (Additional file 5). Hence these findings indicate that ripening related protein may have a dual role during plant defense and fruit ripening. In addition, this study also provides evidence of hormonal cross talks in host chickpea involving SA, JA, ABA and ethylene during Foc1 invasion.

Salicylic acid is also associated with ROS generation. In the present study the proteins related to ROS scavenging showed abrupt increase and downfall in their accumulation level in susceptible plants after infection whereas in resistant plants the accumulation of protein was found to be in a synchronized way and maintained stability (Additional file 5). It may be assumed that the ROS machinery gets activated in both resistant and susceptible plants after infection but acts differently. The sudden generation of ROS and lack of proper ROS scavenging machinery leads to oxidative stress in case of compatible interaction while in case of incompatible interaction they are efficiently detoxified by scavenging machinery. Hence balanced ROS generation in resistant plants act as signaling molecules and communicate downstream defense signals. Previous studies based on transcriptomic profiling indicate the significance of several ROS regulators to act as the initial trigger communicating downstream defense signals [16]. Interestingly the present case study identified similar set of ROS regulating proteins that not only provided correlation between the transcriptomic and proteomic studies, but also highlighted the conservation of ROS components in regulating host defense during Foc1 infection.

The high accumulation of signaling protein like, ABA responsive protein (RAB) only in resistant genotype in present study predicts the involvement of ABA-mediated signaling in plant defense (Additional file 5). ABA responsive protein was reported to be involved in PR-protein induction and disease resistance in other related studies [98]. Besides, the similar accumulation pattern of AGB (Guanine nucleotide binding protein) and annexinD1 in both genotypes after infection further highlighted the role of ABA and calcium in regulating defense signals.

The accumulation of proteins related to energy metabolism, ATP synthesis and degradation, amino acid metabolism, secondary metabolism etc, in both genotypes; although of different levels suggest that in both cases the pathogen targets the primary metabolism of the host (Additional file 5). Resistant plants probably safeguard their essential metabolic elements from the fungal catastrophe while the susceptible plants fail to do so and submit to pathogenic endeavors.

Initially the events of PTI and ETI were thought to be distinct, but recent studies revealed that the components of PTI and ETI overlap [5]. In present study the initial accumulation of ROS scavengers and regulators direct towards possible responses related to PTI. However categorization of other proteins under the categories of PTI and/or ETI could prove to be erroneous without further experimentation. Even then, all these findings as a whole indicate that plant defenses are controlled by complex signaling pathways which are interconnected to each other.

### Correlation between protein and mRNA levels

**Figure 5:**
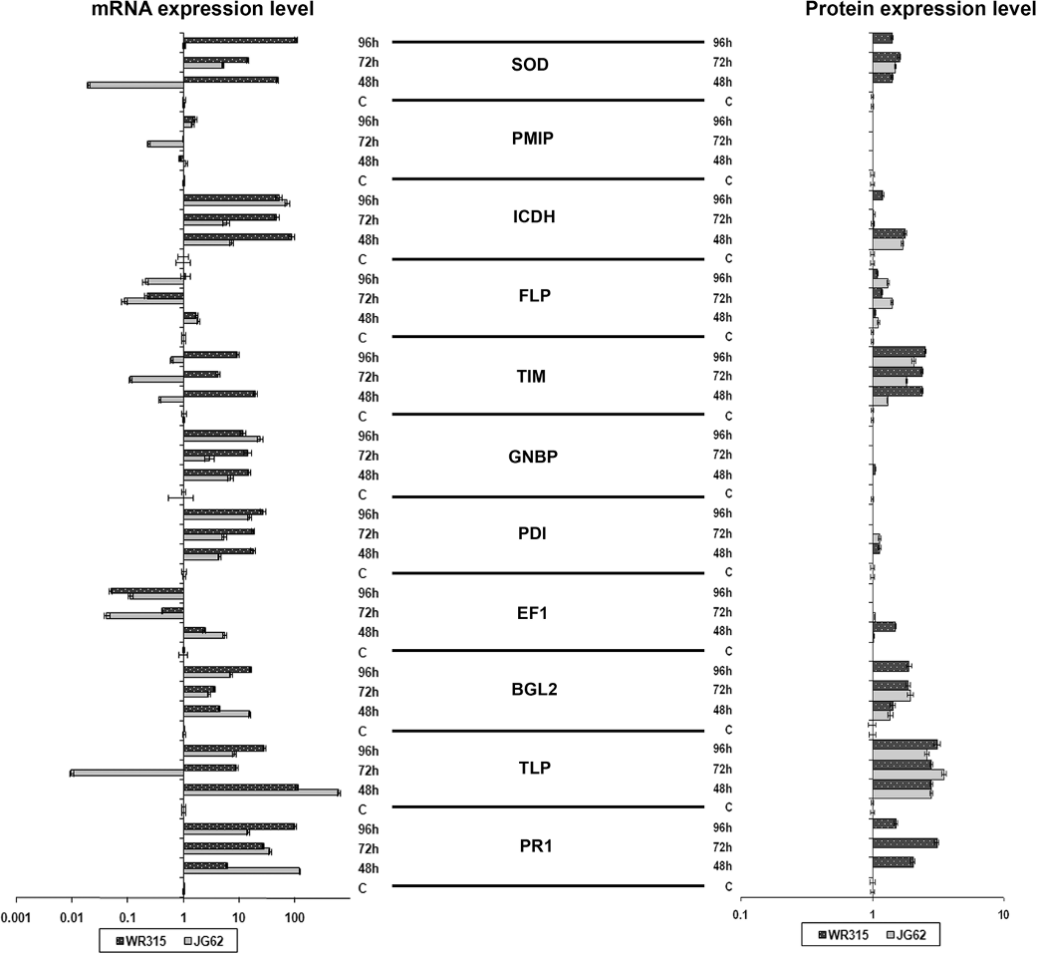
**Comparison of mRNA and protein expression levels of eleven representative genes.** Quantitative real time PCR was performed using gene specific primers (Additional file 2). The log_10_ transformed fold change values (infected/control) of protein spot intensities and mRNA expression level were plotted at different time intervals (48 h, 72 h and 96 h) after infection with Foc1 for both chickpea genotypes. JG62 represented by grey color bars and WR315 represented by black color bars. The proteins selected are SOD (superoxide dismutase), PMIP (Pasma membrane intrinsic protein), ICDH (Isocitrate dehydrogenase), FLP (Fructokinase like protein), TIM (Triose phosphate isomerase), GNBP (Guanine nucleotide binding protein), PDI (Protein disulfide isomerase), EF1 (Elongation factor1), BGL2 (Glucan-endo-1, 3-beta-glucosidase), TLP (Thaumatin like protein), PR1 (Pathogenesis related protein1).

To correlate the protein levels with mRNA levels eleven representative genes corresponding to MS/MS identified proteins were selected and their transcript accumulation versus protein abundance analyzed (Figure 5). The genes of corresponding proteins selected for transcript accumulation were pathogenesis related protein 1(PR1) (sp 145), thaumatin like protein (TLP) (sp 83), glucan-endo-1,3-beta-glucosidase EC 3.2.1.39 (BGL) (sp239), elongation factor 1 (EF1) (sp 275), protein disulfide isomerase (PDI) (sp 102), guanine nucleotide binding protein (GNBP) (sp 233), triose phosphate isomerase EC 5.3.1.1 (TIM) (sp 109), fructokinase like protein (FLP) (sp54), isocitrate dehydrogenase EC 1.1.1.42 (ICDH) (sp 165), plasma membrane intrinsic protein (PMIP) (sp19) and superoxide dismutase EC 1.15.1.1 (SOD) (sp 103). In general the abundance of mRNA differed from that of protein levels suggesting that the fold increment and/or decrement in mRNA accumulation do not correlate with the protein fold changes. Except for PR1, TLP and ICDH, the other eight proteins and their corresponding transcripts showed a similar qualitative trend in their accumulation patterns. However, the profiles did not provide quantitative similarity. PR, TLP and ICDH showed dissimilar patterns suggesting that mRNA and protein levels often exhibit different profiles. Transcript to protein production involves several regulatory factors which are spatially and temporally regulated due to which there are seldom profile matches between mRNA and protein levels. Moreover this disparity between mRNA and protein level might be due to posttranscriptional or posttranslational modifications, complexities of protein expression or presence of multigene families [39]. Similar results were reported in proteomic analysis of strawberry during *Colletotrichum fragariae* infection [99].

## Conclusion

The present study was an attempt to investigate the differential root proteome and identify defense related proteins in chickpea during Foc1 infection. Previous report based on proteome studies involving chickpea Foc5 and root knot nematode *Meloidogyne artiellia* highlighted the presence of several defense responsive proteins [100]. But the difference in pathogenic race is expected to yield some case specific results and hence needs to be studied as an individual case study. The findings of this study suggests that albeit some common proteins are accumulated in response to Foc1 infection in both compatible and/or incompatible chickpea genotypes, but their differential temporal accumulation and regulation probably governs the net outcome of the interaction. The present study highlights the role of several important proteins like PR proteins (PR1, BGL2, TLP), Trypsin protease inhibitors (TPI), ABA responsive protein (RAB18), cysteine proteases (RD19, RD21), methylesterases, 26S proteasome subunits, protein disulphide isomerase (PDIL), ripening related protein (RLP), profilins (PFRs) and albumins and their varied accumulation in susceptible and resistant plants. The functional characterization of these proteins could not only yield important new findings in reevaluating the resistance mechanism of chickpea during Foc1 infection but also help in directing crop improvement programs by using breeding and genetic engineering techniques. Therefore further experiments are necessitated to strengthen the knowledge and understanding through detailed investigations.

### Availability of supporting data

The data sets supporting the results of this article are included within the article and its additional files. The protein and peptide data sets supporting the results are presented in Additional file 5.

## Electronic supplementary material

Additional file 1: **Protein names, abbreviations and TAIR gene IDs.** Table containing list of proteins, their abbreviations used for pathway construction and qRT-PCR and TAIR homologous IDs of the identified proteins used as input for network generation. (XLSX 12 KB)

Additional file 2: **List of primers designed for the qRT-PCR.** List includes the primer pair sequences used for qRT-PCR for identified proteins wth their respective spot IDs. (XLSX 10 KB)

Additional file 3: **Comparative analysis of differentially accumulated protein spots in infected chickpea genotypes.** Includes details of protein yield, average number of spots, variable spots (Quantitative and qualitative) obtained in control and infected chickpea genotypes (JG62 and WR315) at different time points post Foc1 infection. (XLSX 11 KB)

Additional file 4: **Schematic representation of experimental design.** A flow chart depicting the experimental design of Foc1 infected root proteome in chickpea plants. Two weeks old seedlings were infected with Foc1. Root tissues were harvested and 250 μg of proteins were extracted from pooled root tissue to run gels for each time points. The experiments were repeated three times to generate three biological replicates. The gels were stained with coomassie blue and further processed for downstream analyses (In total 72 reproducible 2DE gels were generated). Three technical replicates from three biological replicates were used for PD quest analysis. Differential spots were picked, trypsinized and processed for MALDI- TOF MS and MS/MS. (TIFF 7 MB)

Additional file 5: **Protein spots identified by MALDI-TOF MS AND MS/MS.** Includes details of differential and unique proteins and their peptides identified by MS and MS/MS. The expression pattern of these proteins in control and infected chickpea genotypes (JG62 and WR315) at different time points post Foc1 infection are also illustrated. (XLSX 654 KB)

Additional file 6: **MANOVA Table.** Table includes mean protein spot intensities for identified protein spots for control and infected chickpea cultivars (JG62 and WR315) at different time points upon Foc1 infection. Each value represents mean of three repeated experiments each with three replications. The means followed by the same letters within a row do not differ statistically according to Duncan’s multiple range tests at a 5% probability level. (XLSX 20 KB)

Additional file 7: **Representative cropped gel images of protein spots belonging to different functional categories.** Images show quantitative changes among control and infected plants of both (JG62) and (WR315) chickpea genotypes at different time intervals of 48 h, 72 h, and 96 h after Foc1infection.The number and name indicates the spot identity and name of the proteins mentioned in Additional file 5. Arrow represents the presence of spots. The proteins represented are Pathogenesis related Protein (PR 1), Thaumatin like protein PR- 5b (TLP), Glucan-endo-1,3-beta-glucosidase (BGL2), Trypsin protein inhibitor 3(TrpI-3), Superoxide dismutase (SOD; Mitochondrila manganese SOD), Ascorbate peroxidase (APX), Glutathione S transferase parA(GST), Monodehydro ascorbate reductase (MDAR), Annexin (ANX), ABA-responsive protein (RAB/ABARE), Auxin - induced protein PCNT 115 (Aux ind pro), Triose phosphate isomerase(TIM), Enolase, Isocitrate dehydogenase [NADP] chloroplastic (ICDH), ATP synthase, sub unit D chain (ATPase sub D), S-adenosyl methionine synthetase (SAM), Cysteine proteinase (Cys Pro), Chalcone isomerase(CI), Fructokinase-like protein (FLP), Cytochrome C oxidase subunit 6b-1(COX), Methylesterase1(MER), Adenylate kinase (ADK), 20S proteasome alpha subunit D (20S Prot alpha-D), Protein disulfide-isomerase A6 (PDI), Ripening related protein (RLP/RRP), Germin-like protein (GLP), Profilin-1, Outer plastidial membrane protein porin (Porin), Chain A, Crystal Structure Of A Plant Albumin (Albumin ). (TIFF 5 MB)

Additional file 8: **Network showing the total interaction of different upregulated proteins in chickpea obtained after 48 h, 72 h and 96 h post infection with Foc1.** Green and pink highlighted components represent the upregulated proteins of WR315 plants and JG62 plants respectively obtained after 48h of infection with Foc1. Blue and orange highlighted components represent the upregulated proteins of WR315 plants and JG62 plants respectively obtained after 72 h of infection with Foc1. Yellow highlighted components represent the upregulated proteins of WR315 plants obtained after 96 h of infection with Foc1. Complete names of protein abbreviations are provided in Additional file 1. (TIFF 12 MB)

Additional file 9: **GO classification (biological process and cellular components).** Graphical representation of differentially expressed protein spots in chickpea roots (JG62 and WR315) based on network derived (pathway studio version 7.1 software) gene ontology classification. Graphs represent up regulated and down regulated proteins at different time points under different biological processes and cellular components. (TIFF 1 MB)

Additional file 10: **GO classification (molecular function).** Graphical representation of differentially expressed protein spots in chickpea roots (JG62 and WR315) based on network derived (pathway studio version 7.1 software) gene ontology classification. Graphs represent upregulated and downregulated proteins at different time points under different molecular functions. (TIFF 2 MB)

## Abbreviations

Foc1: *Fusarium oxysporum* f. sp *ciceri* Race 1
2D PAGE: Two-dimensional polyacrylamide gel electrophoresis
MALDI-TOF MS: Matrix-assisted laser desorption/ionization time of flight tandem mass spectrometry
GO: Gene ontology
qRT-PCR: Real-time quantitative reverse transcriptional PCR
PR: Pathogenesis related
PTI: Pattern triggered immunity
ETS: Effector triggered susceptibility
ETI: Effector triggered immunity
FAO: Food and Agriculture Organization
IEF: Isoelectric focusing
MANOVA: Multivariate analysis of variance
DMRT: Duncan’s multiple range test
SA: Salicylic acid
JA: Jasmonic acid
ABA: abscisic acid
NPR1: Non expressor of PR genes1
ACD: Accelerated cell death
MAP kinase: Mitogen activated protein kinase
EDS4: Enhanced disease susceptibility 4
PAD2: Phytoalexin deficient 2
EDR2: Enhanced disease resistance 2
EIL: Ethylene-insensitive3-like
ROS: Reactive oxygen species
GPCRs: G-protein coupled receptors
HR: Hypersensitive response
SAR: Systemic acquired resistance.
