# Supplementary figures and images for: Analysis of root proteome unravels differential molecular responses during compatible and incompatible interaction between chickpea (Cicer arietinum L.) and Fusarium oxysporum f. sp. ciceri Race1 (Foc1)
Source: BMC Genomics. 2014 Nov 3;15(1):949. doi: 10.1186/1471-2164-15-949 (PMC4237293; doi:10.1186/1471-2164-15-949)

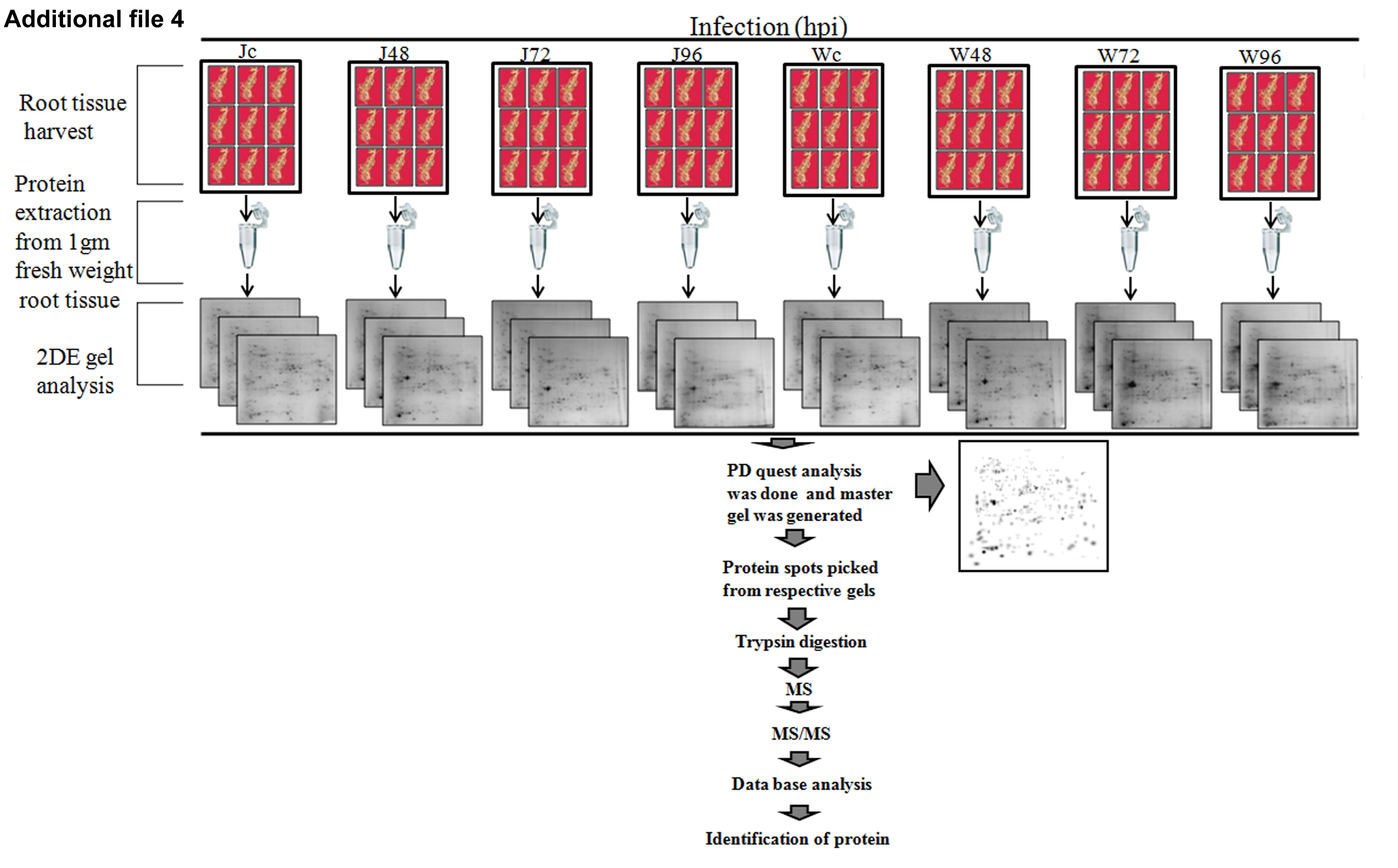

Supplement: Supplementary file 4 — Additional file 4: Schematic representation of experimental design. A flow chart depicting the experimental design of Foc1 infected root proteome in chickpea plants. Two weeks old seedlings were infected with Foc1. Root tissues were harvested and 250 μg of proteins were extracted from pooled root tissue to run gels for each time points. The experiments were repeated three times to generate three biological replicates. The gels were stained with coomassie blue and further processed for downstream analyses (In total 72 reproducible 2DE gels were generated). Three technical replicates from three biological replicates were used for PD quest analysis. Differential spots were picked, trypsinized and processed for MALDI- TOF MS and MS/MS. (TIFF 7 MB) [file 12864_2014_6658_MOESM4_ESM.tiff]

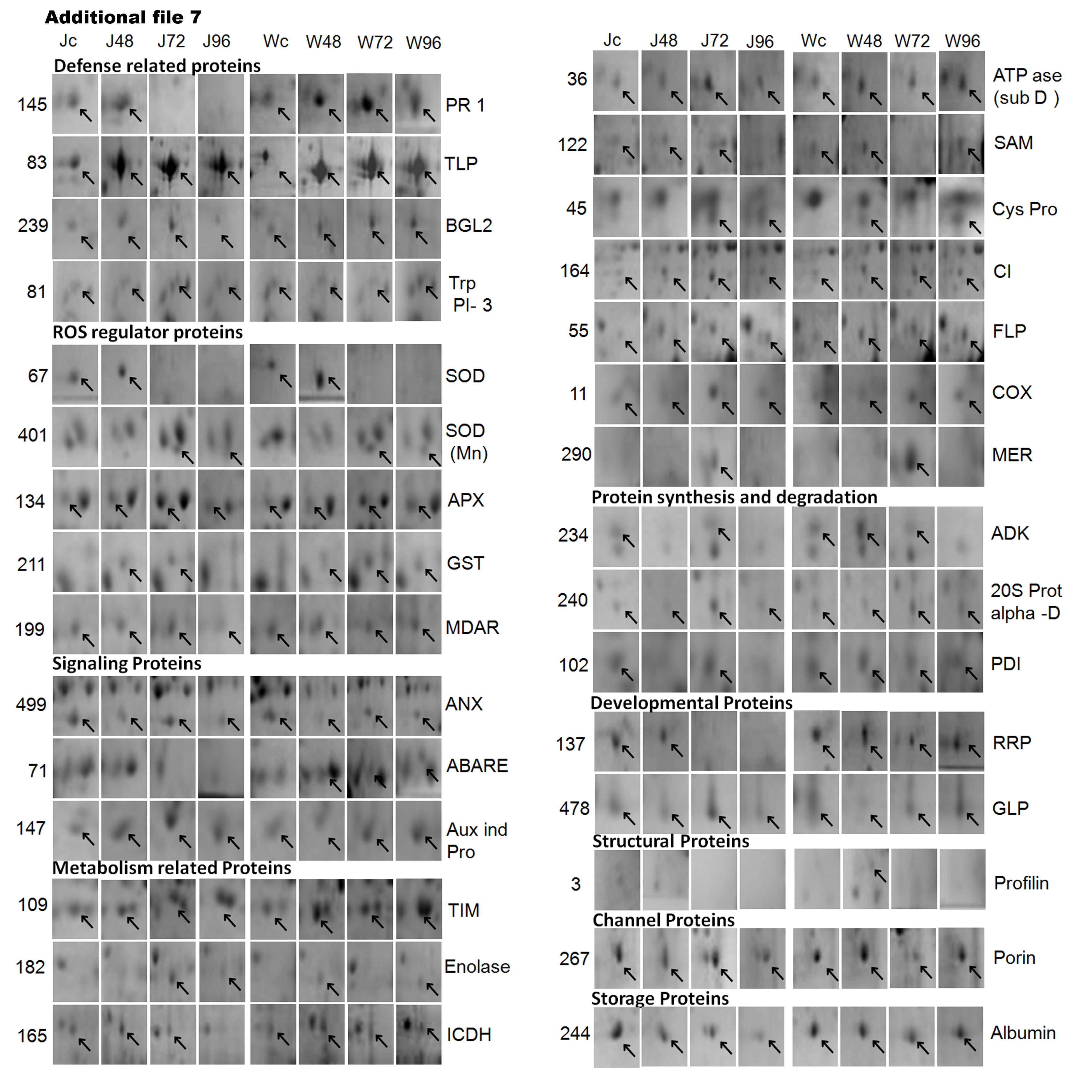

Supplement: Supplementary file 7 — Additional file 7: Representative cropped gel images of protein spots belonging to different functional categories. Images show quantitative changes among control and infected plants of both (JG62) and (WR315) chickpea genotypes at different time intervals of 48 h, 72 h, and 96 h after Foc1infection.The number and name indicates the spot identity and name of the proteins mentioned in Additional file 5. Arrow represents the presence of spots. The proteins represented are Pathogenesis related Protein (PR 1), Thaumatin like protein PR- 5b (TLP), Glucan-endo-1,3-beta-glucosidase (BGL2), Trypsin protein inhibitor 3(TrpI-3), Superoxide dismutase (SOD; Mitochondrila manganese SOD), Ascorbate peroxidase (APX), Glutathione S transferase parA(GST), Monodehydro ascorbate reductase (MDAR), Annexin (ANX), ABA-responsive protein (RAB/ABARE), Auxin - induced protein PCNT 115 (Aux ind pro), Triose phosphate isomerase(TIM), Enolase, Isocitrate dehydogenase [NADP] chloroplastic (ICDH), ATP synthase, sub unit D chain (ATPase sub D), S-adenosyl methionine synthetase (SAM), Cysteine proteinase (Cys Pro), Chalcone isomerase(CI), Fructokinase-like protein (FLP), Cytochrome C oxidase subunit 6b-1(COX), Methylesterase1(MER), Adenylate kinase (ADK), 20S proteasome alpha subunit D (20S Prot alpha-D), Protein disulfide-isomerase A6 (PDI), Ripening related protein (RLP/RRP), Germin-like protein (GLP), Profilin-1, Outer plastidial membrane protein porin (Porin), Chain A, Crystal Structure Of A Plant Albumin (Albumin ). (TIFF 5 MB) [file 12864_2014_6658_MOESM7_ESM.tiff]

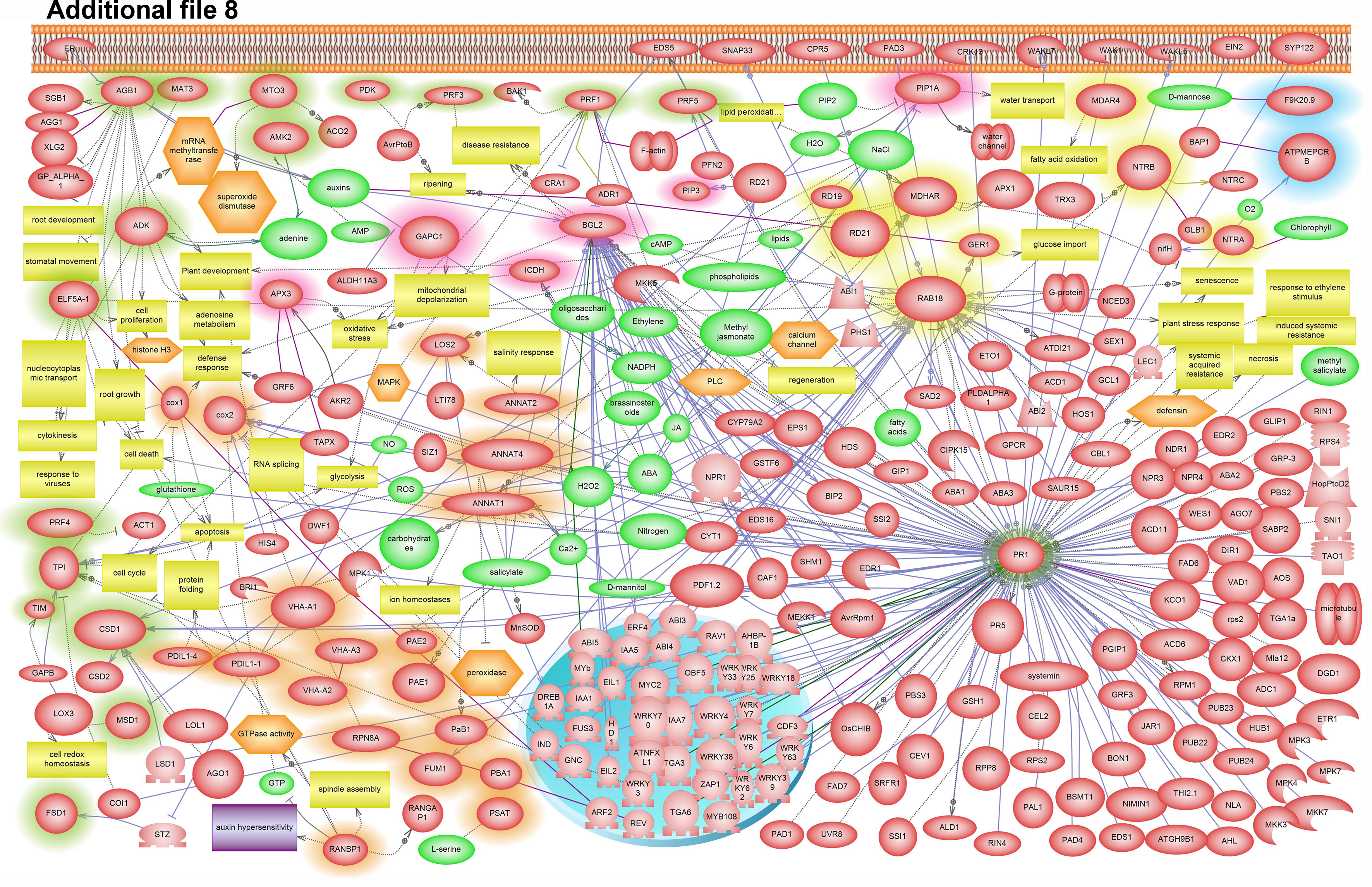

Supplement: Supplementary file 8 — Additional file 8: Network showing the total interaction of different upregulated proteins in chickpea obtained after 48 h, 72 h and 96 h post infection with Foc1. Green and pink highlighted components represent the upregulated proteins of WR315 plants and JG62 plants respectively obtained after 48h of infection with Foc1. Blue and orange highlighted components represent the upregulated proteins of WR315 plants and JG62 plants respectively obtained after 72 h of infection with Foc1. Yellow highlighted components represent the upregulated proteins of WR315 plants obtained after 96 h of infection with Foc1. Complete names of protein abbreviations are provided in Additional file 1. (TIFF 12 MB) [file 12864_2014_6658_MOESM8_ESM.tiff]

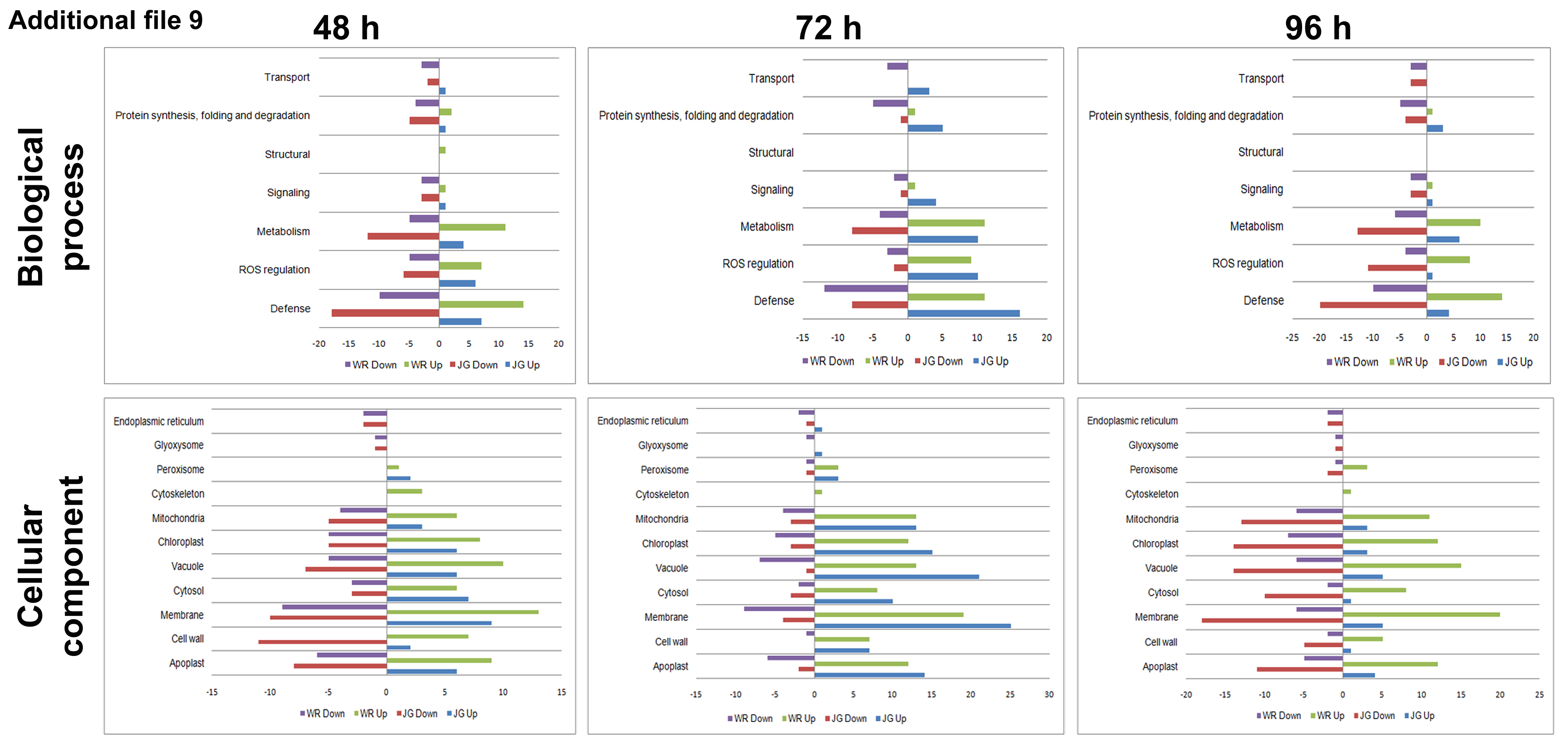

Supplement: Supplementary file 9 — Additional file 9: GO classification (biological process and cellular components). Graphical representation of differentially expressed protein spots in chickpea roots (JG62 and WR315) based on network derived (pathway studio version 7.1 software) gene ontology classification. Graphs represent up regulated and down regulated proteins at different time points under different biological processes and cellular components. (TIFF 1 MB) [file 12864_2014_6658_MOESM9_ESM.tiff]

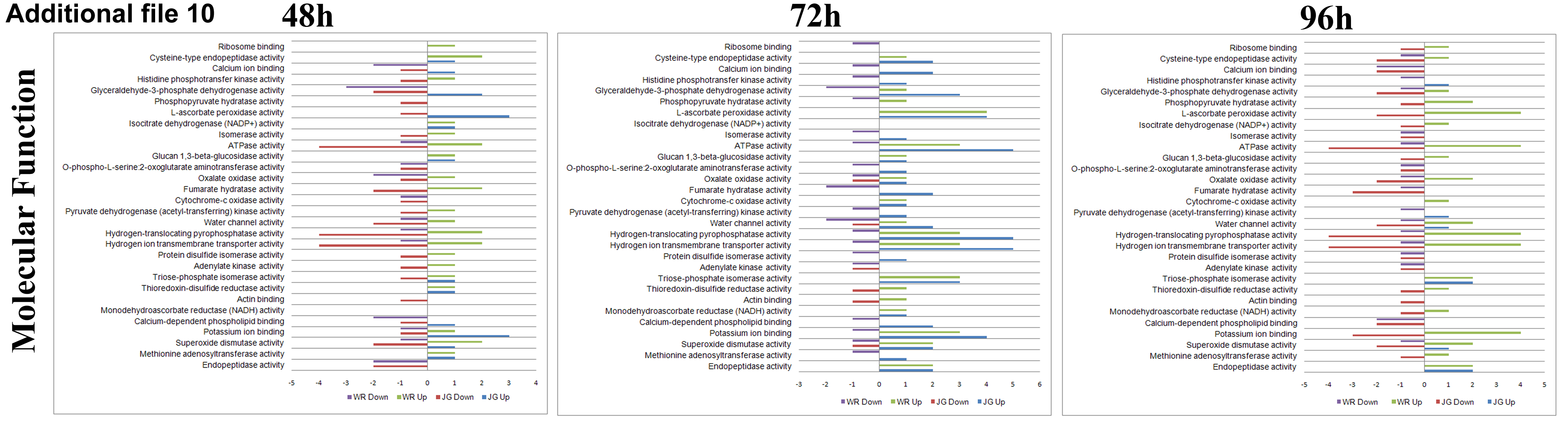

Supplement: Supplementary file 10 — Additional file 10: GO classification (molecular function). Graphical representation of differentially expressed protein spots in chickpea roots (JG62 and WR315) based on network derived (pathway studio version 7.1 software) gene ontology classification. Graphs represent upregulated and downregulated proteins at different time points under different molecular functions. (TIFF 2 MB) [file 12864_2014_6658_MOESM10_ESM.tiff]
